# Alterations in gamma-aminobutyric acid and glutamate neurotransmission linked to intermittent theta-burst stimulation in depression: a sham-controlled study

**DOI:** 10.1038/s41398-025-03371-x

**Published:** 2025-04-08

**Authors:** Linda Steinholtz, Robert Bodén, Anders Wall, Mark Lubberink, David Fällmar, Jonas Persson

**Affiliations:** 1 Department of Medical Sciences, Uppsala University, Uppsala, Sweden; 2 PET-Centre, Uppsala University Hospital, Uppsala, Sweden; 3 Department of Surgical Sciences, Molecular Imaging and Medical Physics, Uppsala University, Uppsala, Sweden; 4 Department of Surgical Sciences, Neuroradiology, Uppsala University, Uppsala, Sweden

**Keywords:** Depression, Pathogenesis, Bipolar disorder

## Abstract

Gamma-aminobutyric acid (GABA) and glutamate are implicated in the antidepressant effects of repetitive transcranial magnetic stimulation (rTMS), though findings from magnetic resonance spectroscopy (MRS) are inconsistent. Furthermore, the relationship between GABA_A_-receptor availability and rTMS outcomes remains largely unexplored. In this study, GABA and glutamate levels in the dorsal anterior cingulate cortex (dACC) were measured using a ^1^H-MRS MEGA-PRESS sequence in 42 patients with bipolar or unipolar depression, both before and after a sham-controlled, double-blind clinical trial involving intermittent theta-burst stimulation (iTBS) over the dorsomedial prefrontal cortex. A subset of 28 patients also underwent [^11^C]flumazenil positron emission tomography (PET) to measure whole-brain GABA_A_-receptor availability and mean receptor availability in the nucleus accumbens and dACC. Depressive symptoms were assessed using the self-rated Montgomery Åsberg Depression Rating Scale (MADRS-S). The results indicated no significant changes in neurotransmitter levels or GABA_A_-receptor availability post-iTBS in either the active or sham conditions. However, changes in MADRS-S scores after active iTBS were positively correlated with changes in GABA levels in the dACC (r(13) = 0.54, *p* = *0.04*) and baseline GABA_A_-receptor availability in the nucleus accumbens (r(11) = 0.66, *p* = *0.02*). These correlations were absent in the sham group. The findings suggest that a reduction in GABA within targeted frontostriatal circuits can be part of the antidepressant mechanism of iTBS, challenging previous research. Additionally, they indicate a potential predictive role for frontostriatal GABA_A_-receptor availability in the treatment of depression using dorsomedial prefrontal iTBS.

## Introduction

Repetitive transcranial stimulation (rTMS) has emerged as an effective treatment for depressive episodes [1]. However, the mechanisms through which rTMS exerts its effects remain elusive. In addition to regulating activity in the stimulated brain areas, antidepressant rTMS protocols also influence activity in interconnected regions implicated in depression, such as the anterior cingulate cortex (ACC) and the nucleus accumbens [2, 3]. Furthermore, it is suggested that the mechanism of rTMS may affect the metabolism of gamma-aminobutyric acid (GABA) and glutamate, the main inhibitory and excitatory neurotransmitters in the central nervous system [4, 5].

Dysfunction in GABA has been implicated in depressive episodes, as supported by a growing body of evidence [6–9]. Magnetic resonance spectroscopy (MRS) is a rapidly evolving method that allows for in vivo quantification of metabolite levels in the brain. By employing advanced techniques, such as J-editing, it is possible to quantify even molecules of very low abundance in the brain, such as GABA [10]. MRS meta-analyses have pointed to lower GABA levels in the medial frontal cortex, specifically the ACC, in depressed patients compared to healthy controls [11, 12], further supporting the role of GABA dysfunction in depression.

A few studies have examined GABA levels in the context of rTMS treatments for depression; however, the findings have been inconsistent. Two studies reported an increase in GABA concentrations in the left dorsolateral prefrontal cortex (dlPFC) and medial prefrontal cortex (mPFC), noting that individuals who responded to treatment exhibited a greater increase in GABA than non-responders [13, 14]. Another study observed an increase in GABA levels in the mPFC following intermittent theta burst stimulation (iTBS), a patterned high-frequency form of rTMS, applied over the left dlPFC; however, this increase was not associated with an antidepressant effect [15]. In contrast, two other studies found no changes in GABA levels in the left dlPFC after rTMS, irrespective of symptom improvement. These latter studies also evaluated baseline GABA levels, which were not predictive of clinical outcomes [16, 17].

Not much is known about the GABA_A_ receptor in the context of depressive states, and even less about its role in the mechanism of rTMS. A single photon emission computed tomography (SPECT) study using [^123^I]iomazenil to assess in vivo GABA_A_-receptor availability could not detect any difference between patients with depression and healthy controls [18]. Similarly, in a study using [^11^C]flumazenil positron emission tomography (PET), we found no difference in GABA_A_-receptor availability in depressed patients compared to healthy individuals; this study included the same patients as those in the current investigation [19]. However, an earlier study using [^11^C]flumazenil PET found reduced binding to GABA_A_ receptors in the bilateral temporal cortices of depressed patients compared with healthy controls [20]. While there have been no prior studies on GABA_A_-receptor availability after rTMS for depression, a study using [^123^I]iomazenil SPECT demonstrated a widespread increase in cortical GABA_A_ receptor binding in depressed patients following successful electroconvulsive therapy [21].

Closely related to the GABA dysfunction hypothesis is the suggestion of alterations in glutamate neurotransmission in depressive episodes [6, 22]. Glutamate is intrinsically linked to GABA due to their shared metabolic pathways and the essential balance between their opposing functions [6]. A meta-analysis indicated a decrease in Glx (a compound measure of glutamate and glutamine) in the mPFC of patients with major depressive disorder compared to controls. However, there was no significant difference in glutamate or glutamine separately, and the effect size was small, with large heterogeneity across studies [23].

Studies of glutaminergic metabolites after rTMS are limited, with the results varied. One study found that responders had significantly lower baseline glutamate levels [24], whereas baseline Glx has been both positively and negatively correlated with treatment outcomes [16, 17]. While several studies have found no post-rTMS changes in glutamate or Glx in pooled samples [14, 17, 24], there are findings of symptom improvement being correlated to both increased glutamate [24] and reduced Glx levels [17].

In summary, there is evidence supporting altered GABA and glutamate function in depressive episodes. However, it remains unclear whether antidepressant rTMS treatment affects GABA and glutamate, and whether the clinical effects of rTMS are dependent on baseline neurotransmitter levels or changes in these neurotransmitters following treatment. Sham-controlled studies, which could provide information on this matter, are still very sparse.

### Aims

This study aimed to examine changes in GABA and glutamate levels in the dorsal ACC (dACC), as well as changes in the availability of GABA_A_ receptors, following active versus sham iTBS for depression. Additionally, the study assessed whether such changes were associated with alterations in depressive symptoms after active and sham iTBS. Finally, we investigated whether baseline measurements of GABA levels, glutamate levels, and GABA_A_-receptor availability were associated with clinical outcomes following iTBS.

## Methods

### Participants

This study included 42 patients from a clinical trial of iTBS at the psychiatric clinic at Uppsala University Hospital [25]. The inclusion criteria were being between 18 and 59 years old, having an ongoing bipolar or unipolar depressive episode verified by the Mini International Neuropsychiatric Interview (M.I.N.I.) (Swedish translation of version 6.0.0), having had unchanged psychiatric medication the preceding month, and scoring ≤40 points on the Motivation and Pleasure Scale-Self-Report [26, 27]. Exclusion criteria included epilepsy diagnosis, the presence of ferromagnetic or other metal implants, benzodiazepine treatment, substance use disorder (except for nicotine and caffeine), and pregnancy. Any pharmacotherapy was kept constant throughout the study period. The Research Ethical Review Board in Uppsala provided ethical approval for the study. Written informed consent was obtained from all participants, and the study was conducted in accordance with the Declaration of Helsinki.

### Rating scales

Upon inclusion, patients were assessed using the Brief Psychiatric Rating Scale – extended (BPRS-E). The BPRS-E is a semi-structured interview comprising 24 items that measure a broad range of psychiatric symptoms [28]. The affective subscore consists of the items ‘depression’, ’suicidality’, and ‘guilt’ and ranges from 3 to 21, with a higher score indicating more severe symptomatology [29].

To evaluate the severity and changes in depression symptoms, the self-rated version of the Montgomery Åsberg Depression Rating Scale (MADRS-S) was used [30]. The MADRS was developed specifically to be sensitive to the effects of antidepressant treatments [31].

### Experimental design and procedure

The patients participated in a sham-controlled, double-blind clinical trial of iTBS over the dorsomedial prefrontal cortex (dmPFC), given twice daily over 10–15 consecutive weekdays. The study used block randomisation with block sizes of 6 and 8 in random order. Below is a summary of the treatment procedure; a comprehensive description is found elsewhere [32].

Treatments were delivered using a Magpro X100 (MagVenture, Farum, Denmark) with a Cool D-B80 coil. The coil was a combined active/placebo coil with two identical sides that were positioned tangentially to the scalp, with the handle pointing to the right side of the patient. The resting motor threshold (rMT) was defined as the lowest intensity needed to elicit a visually observable twitching in either foot in 50% of the trials, targeting the medial primary motor cortex.

An MRI-based neuronavigational system (TMS navigator, Localite, Bonn, Germany) was utilised to target the dmPFC at the Montreal Neurological Institute (MNI) coordinates x = 0, y = 30, z = 30 [33]. The active treatment followed a modified version of the previously described iTBS protocol [34, 35]. It consisted of 20 trains of right-left stimulation and 20 trains of left-right stimulation at 90% of rMT. Each train contained ten bursts at five Hertz, and each burst comprised three biphasic pulses at 50 Hertz. An eight-second pause separated each two-second stimulation train. Two identical treatment sessions were conducted, with a 15-minute break in between, to accelerate treatment [36, 37]. A total of 1200 pulses were given per session, totalling 2400 pulses per day. If less than 50% of the trains reached the target intensity on any given treatment day, the treatment course was prolonged with one extra day, up to a maximum of 15 days.

All patients had two transcutaneous electrical nerve stimulation (TENS) electrodes placed medially on the forehead under the coil. For the sham treatment, the shielded side of the coil was placed against the head, preventing the magnetic pulses from the stimulation protocol from reaching the patient. Instead, electric currents were delivered through the TENS electrodes, synchronised with the magnetic pulses, to mimic the sensation of magnetic stimulation.

One weekday before the treatment onset and again four weeks later, all patients underwent magnetic resonance imaging, including MRS, and completed the MADRS-S. A sub-sample of 28 patients also underwent PET.

### Magnetic resonance imaging and spectroscopy

The investigations were conducted using a 3 Tesla scanner (Achieva dStream, Philips Healthcare, Best, The Netherlands) equipped with a 32-channel head coil. T1-weighted structural images were obtained using a 3D turbo TFE sequence (TR/TE = 8.2/3.8 ms, flip angle = 8°, field of view = 256 × 256 mm, and spatial resolution = 1 × 1 × 1 mm). All MRI images were assessed by a specialist in neuroradiology to rule out any focal lesions or abnormal anatomy.

MRS was performed using the J-difference Mescher-Garwood spectral editing technique, implemented within a point-resolved spectroscopy sequence (MEGA-PRESS) [10]. MEGA-PRESS is developed for the quantification of GABA, but it also gives a reliable estimate of glutamate using the OFF spectra [38]. The experimental parameters were: TR/TE 2000/68 ms, spectral bandwidth 2000 Hz, 1024 points, and phase cycling 4. For each condition (ON and OFF), 160 averages were obtained. The data were gathered in groups of 40, each starting with an unsuppressed water line, followed by four consecutive pairs of water-suppressed transients in the ON and OFF conditions. The water lines were used for magnetic field drift correction and to update the carrier frequency of radiofrequency pulses for each group.

**Fig. 1:**
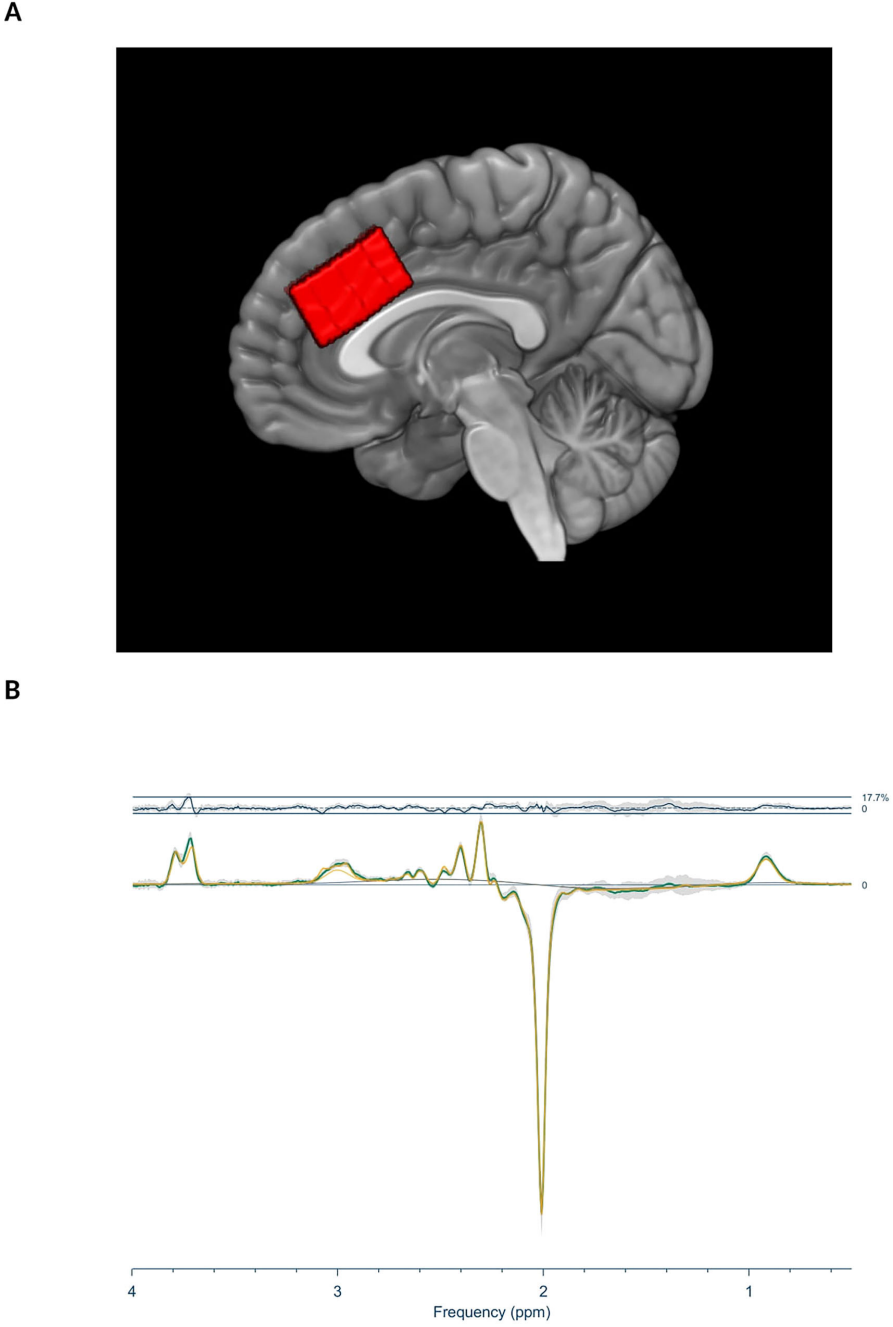
Voxel localisation and difference spectra at baseline. **A** Illustration of voxel placement. **B** Mean difference (ON-OFF) spectra (green) with standard deviation (grey), mean residual (above) and mean fit (yellow) from the baseline acquisitions.

A voxel of interest measuring 40 × 40 × 20 mm (left-right x anterior-posterior x feet-head directions) was positioned along the bilateral cingulate gyrus, with its inferior border coinciding with the superior border of the corpus callosum and its anterior border in line with the most anterior part of the corpus callosal genu (Fig. 1).

Pre-processing, modelling, and quantification of the MEGA-PRESS spectra were performed using Osprey 2.5.0 [39] in Matlab R2023b. The processing steps included alignment with the probabilistic spectral registration algorithm and eddy current correction. After phase and frequency correction, the individual averaged spectra were visually inspected to detect irregularities. The spectra were fitted with the Osprey linear combination method using the default basis set, with a separate fitting for difference and OFF spectra, and a baseline knot spacing of 0.55 ppm [40]. T1-weighted images were utilised for tissue segmentation using the unified segmentation algorithm implemented in Statistical Parametric Mapping 12 (SPM12; Wellcome Trust Centre for Neuroimaging Institute of Neurology, University College of London, UK). This process allowed for the calculation of the fractions of grey matter, white matter, and cerebrospinal fluid within the MRS voxel.

The difference (ON-OFF) and OFF spectra were used for GABA and glutamate quantification, respectively (Fig. 1). The levels were estimated using alpha-corrected tissue water as the reference [41, 42]. Quality metrics for the MRS signal were determined by calculating the average signal-to-noise ratio (SNR) and the mean linewidth of creatine. Relative residuals were calculated by comparing the amplitude of the residuals to the standard deviation (SD) of the noise, serving as an indicator of the goodness of fit.

### Positron emission tomography

The measurements were performed using a Discovery MI PET/computed tomography (CT) system (GE Healthcare, Waukesha, WI), beginning with a low-dose CT scan (120 kV, 10–20 mA, noise index 170) for attenuation correction of the PET data. Dynamic PET acquisition (4 × 15 s, 4 × 60 s, 2 × 150 s, 2 × 300 s, 2 × 600 s, totalling 40 min) started simultaneously as the participant received a controlled intravenous bolus injection of [^11^C]flumazenil (2–4 MBq/kg body weight) [43]. Image reconstruction was performed using time-of-flight ordered subsets expectation maximisation with 3 iterations and 34 subsets, including resolution recovery, and a 3-mm Gaussian post-processing filter. The [^11^C]flumazenil synthesis was conducted as previously described [43].

VOIager 4.0.7 (GE Healthcare) software was used to correct for within-scan movement across the dynamic PET frames. Using SPM8, the T1-weighed MRI images were co-registered to a summation of the first 5 minutes of the PET scan and segmented into grey and white matter. Parametric images of non-displaceable binding potential (BP_ND_), an estimate of GABA_A_-receptor availability, were derived through a basis function implementation of the simplified reference tissue method (SRTM), using the centrum semiovale white matter as the reference region [44–46]. This region was acquired by removing the two outer layers of voxels of the cranial half of the subjects’ white matter segmentation images. This resulted in an average reference region volume of 4.4 ± 1.4 cm^3^ across subjects. By projecting the reference region across all frames of the dynamic PET scan, a time-activity curve for the reference tissue could be generated.

Additional image pre-processing was carried out using SPM12 in MATLAB 2019b, involving the co-registration of BP_ND_ images to the T1-weighted image. Tissue classification, bias correction, and spatial normalisation of the T1-weighted images were performed using the unified segmentation algorithm implemented in SPM12. Ultimately, a Gaussian kernel with a full-width at half maximum (FWHM) of 8 × 8 × 8 mm was used to smooth the BP_ND_ images.

### Volumes of interest

The first volume of interest for BP_ND_ was the voxel used in spectroscopy. The grey and white matter segments from the tissue segmentation during the Osprey process were used to define a mask for each participant, corresponding to the MRS voxel. The second volume of interest was the nucleus accumbens (bilateral), defined using the CIC atlas [47]. To make individual masks, the inverse deformation fields from the earlier pre-processing were used to adjust the atlas region into the subject space for each participant. Using these masks, the individual mean BP_ND_ within the volumes of interest could be extracted at each time point.

### Statistical methods

Descriptive data were tabulated, and group differences were analysed using Student’s t-test for normally distributed data with equal variance, the Mann-Whitney U test for non-normal data, and the Chi^2^ test for categorical data. Mean BP_ND_ and neurotransmitter levels at baseline (pre) and post-treatment were compared for active and sham iTBS, respectively, using a paired samples Student’s t-test when assumptions were met and Wilcoxon’s signed-rank test when they were not. Delta-values for mean BP_ND_ and neurotransmitter levels, defined as the difference between post-treatment and pre-treatment values, were compared between the active and sham treatment groups using the independent samples Student’s t-test when the assumptions for the test were met; otherwise, the Mann-Whitney U test was used.

Differences in the pre-post treatment change of BP_ND_ between treatment arms were also assessed on a whole brain voxel-wise basis. Delta-value PET images, derived from the differences in BP_ND_ observed in the parametric PET images before and after treatment, were entered into a voxel-wise General Linear Model in SPM, with the treatment groups as separate regressors. F-contrasts were defined to assess voxels where BP_ND_ differed between sham and active treatment. Voxels were considered significant at a cluster threshold of p_FWE_ < 0.05, using a cluster-forming threshold of p < 0.001, uncorrected.

The baseline and delta values of glutamate levels, GABA levels, and mean BP_ND_ were also correlated with changes in MADRS-S scores using the Pearson correlation coefficient separately for each treatment group.

Unless specified otherwise, tests were two-tailed with an alpha level of 0.05. The analyses were performed using JASP 0.18.1 [48] and SPM12 in MATLAB 2019b.

## Results

One participant lacked pre-treatment MRS measurements. For five participants, baseline neurotransmitter levels could not be estimated because of missing water reference. In four cases, this was due to software problems during acquisition, and in one case, it was due to poor quality. Among the post-treatment MRS measurements, two participants had missing MRS data, and two lacked a water reference. In the PET sub-sample, four participants had missing post-treatment PET data.

The active and sham groups did not differ significantly in age or sex distribution. Median MADRS-S scores did not differ between the groups, either before or after treatment (Table 1). Mean neurotransmitter levels and BP_ND_ values are depicted in Table 2. Baseline values did not differ between groups. GABA and glutamate levels remained stable after treatment, regardless of the treatment arm. Similarly, the mean BP_ND_ did not change in the specified volumes of interest. Delta values did not differ between active and sham treatments (Table 3).

**Table 1:** Demographic and clinical data.

	Whole sample				PET sub-sample			
	Active (n = 22)	Sham (n = 20)		*p*	Active (n = 14)	Sham (n = 14)		*p*
Age (years), median (q1, q3)	27 (23, 36)	27 (22, 33)	U = 238	0.66	26 (23, 34)	26 (21, 34)	U = 108	0.66
Range (years)	18–54	18–48			19–54	18–48		
Men/women, n	12/10	9/11	χ^2^ = 0.38	0.54	8/6	7/7	χ^2^ = 0.14	0.70
BPRS-E affective score pre, median (q1, q3)	14 (11, 16)	13 (11, 15)	U = 259	0.33	14 (12, 15)	11 (9, 14)	U = 133	0.11
MADRS-S pre, median (q1, q3)	30 (24, 36)	32 (24, 34)	U = 232	0.78	32 (26, 38)	26 (23, 34)	U = 122	0.28
MADRS-S post, median (q1, q3)	27 (14, 34)	26 (17, 31)	U = 202	0.86	27 (14, 34)	26 (16, 32)	U = 90	1
Bipolar depression, n	2	1			1	1		
Pharmacotherapy:								
Antidepressants, n (UP/BP)	17/2	17/0			11/1	11/0		
Antipsychotics, n (UP/BP)	3/1	4/0			2/1	2/0		
Antiepileptics, n (UP/BP)	2/1	2/0			1/1	0/0		

*PET* positron emission tomography, *n* number of participants, *q* quartile, *BPRS-E* brief psychiatric rating scale – extended, *MADRS-S* Montgomery Åsberg depression rating scale – self-rated, *U* Mann-Whitney U test, *χ*^*2*^ Chi-square test, *UP* unipolar depression, *BP* bipolar depression.

**Table 2:** Comparisons of gamma-aminobutyric acid (GABA) levels, glutamate levels, and non-displaceable binding potentials (BP_ND_) before and after active resp. sham treatment with intermittent theta-burst stimulation (iTBS), and between treatment modalities at baseline.

	Active iTBS		Pre vs. post		Sham iTBS		Pre vs. post		*Active vs. sham pre*	
	pre	post			pre	post				
*Neurotransmitter levels, i.u*.	(n = 17)	(n = 19)		*p*	(n = 19)	(n = 19)		*p*		*p*
GABA, mean (SD)	2.09 (0.41)	2.30 (0.59)^b^	z = −1.53	0.14	2.36 (0.65)	2.26 (0.66)	t = 0.37	0.72	U = 104	0.07
Glutamate, mean (SD)	14.60 (2.32)	13.55 (2.32)	t = 0.92	0.37	13.16 (3.04)	13.15 (2.08)	t = 0.59	0.56	t = 1.59	0.12
*[*^*11*^*C]flumazenil BP*_*ND*_	(n = 14)	(n = 11)		*p*	(n = 14)	(n = 13)		*p*		
MRS voxel, mean (SD)	4.01 (0.54)^a, b^	3.97 (0.34)	z = −0.66	0.56	4.10 (0.47)	4.00 (0.36)	t = 0.42	0.68	U = 67	0.26
Nucleus accumbens, mean (SD)	8.15 (1.18)	8.74 (1.33)	t = −1.16	0.27	8.77 (1.11)	8.66 (1.03)	t = 0.26	0.80	t = −1.42	0.17

*i.u*. institutional units, *n* number of participants, *SD* standard deviation, *MRS* magnetic resonance spectroscopy, *z* Wilcoxon’s signed-rank test; *t* Student’s t-test; *U* Mann-Whitney U test.

^a^n = 13

^b^median (IQR), since Shapiro-Wilk’s test p < 0.05, suggesting deviation from normality.

**Table 3:** Comparisons of change in gamma-aminobutyric acid (GABA) levels, glutamate levels, and non-displaceable binding potentials (BP_ND_) between active and sham treatment with intermittent theta-burst stimulation (iTBS).

	Active iTBS change pre-post	Sham iTBS change pre-post	*Student’s t-test*	
*Neurotransmitter levels, i.u*.	(n = 15)	(n = 18)	t	*p*
GABA, mean (SD)	0.31 (0.75)	−0.09 (1.05)	1.23	0.23
Glutamate, mean (SD)	−0.54 (2.27)	−0.34 (2.43)	−0.24	0.81
			***Mann-Whitney U test***	
*[*^*11*^*C]flumazenil BP*_*ND*_	(n = 11)	(n = 13)	U	*p*
MRS voxel, mean (SD)^a^	0.05 (0.26)^b^	−0.06 (0.52)	64	0.98
Nucleus accumbens, mean (SD)	0.06 (0.36)^c^	−0.06 (0.85)	83	0.53

*i.u*. institutional units, *n* number of participants, *SD* standard deviation, *MRS* magnetic resonance spectroscopy.

^a^Levene’s test p < 0.05, suggesting unequal variance.

^b^n = 10

^c^median (IQR), since Shapiro-Wilk’s test p < 0.05, suggesting deviation from normality.

The voxel-wise analysis did not reveal any clusters with significant differences in BP_ND_ changes between the active and sham treatments.

**Fig. 2:**
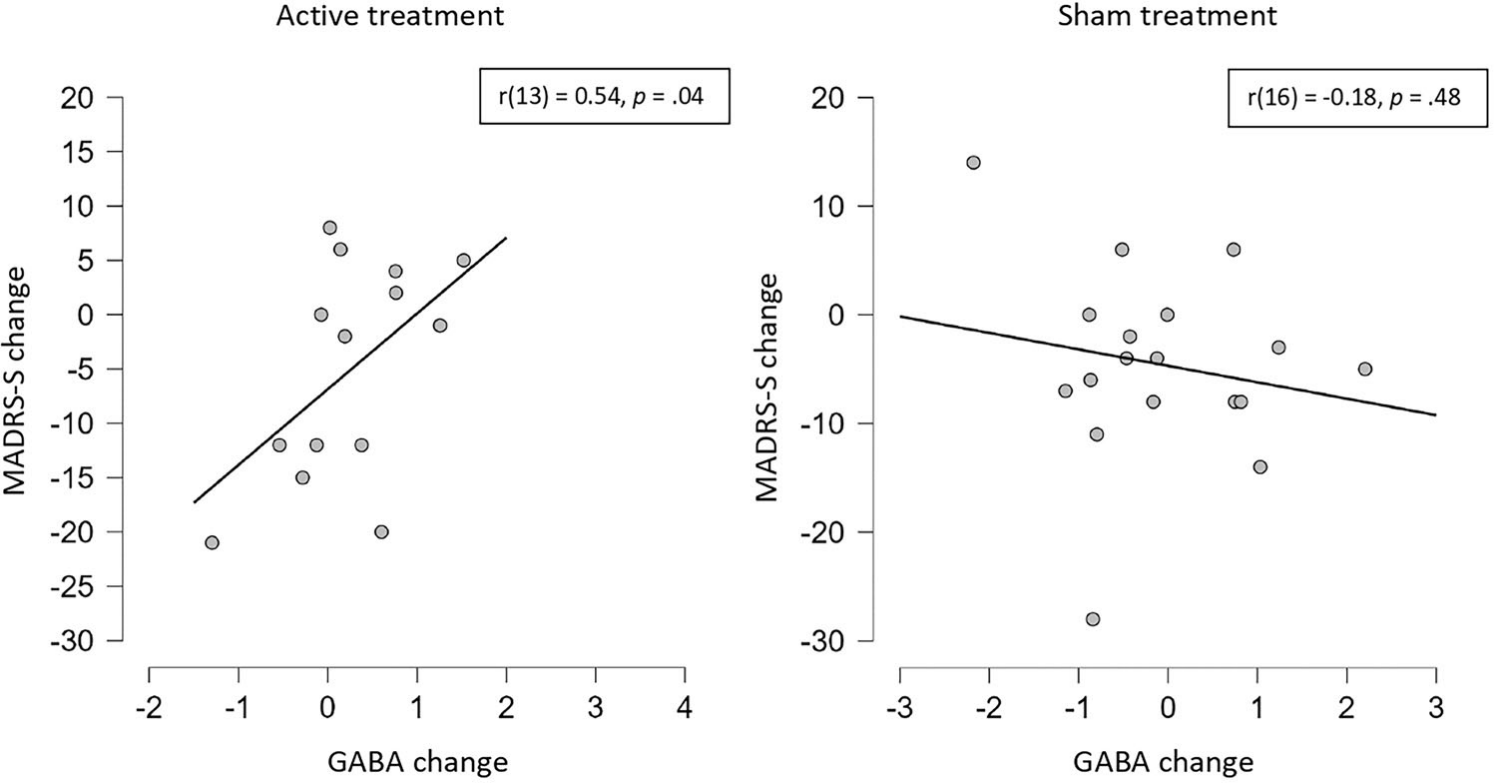
Correlations between change in GABA levels (i.u.) in the dorsal anterior cingulate cortex and change in MADRS-S after active and sham treatment. MADRS-S: Montgomery Åsberg Depression Rating Scale – self-rated; GABA: gamma-aminobutyric acid, i.u: institutional units.

**Fig. 3:**
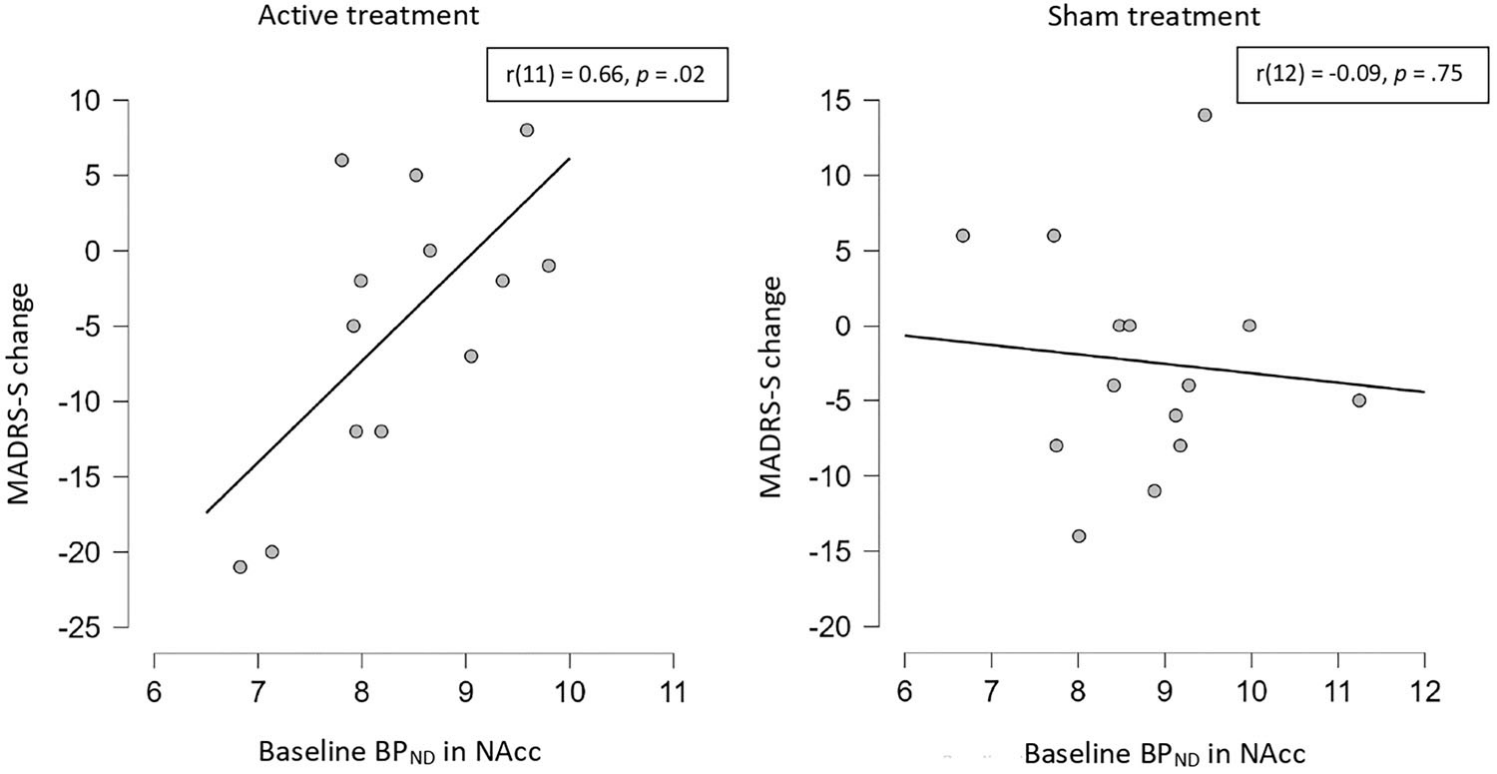
Correlations between baseline BP_ND_ for GABA in nucleus accumbens and change in MADRS-S after active and sham treatment. MADRS-S: Montgomery Åsberg Depression Rating Scale – self-rated, BP_ND_: non-displaceable binding potential, GABA: gamma-aminobutyric acid, NAcc: nucleus accumbens.

In the active treatment group, changes in GABA levels in the dACC were positively related to changes in MADRS-S scores (Fig. 2). No such correlation was seen between changes in glutamate levels and MADRS-S change. There was also a positive correlation between baseline BP_ND_ in the nucleus accumbens and changes in MADRS-S scores (Fig. 3) within the active treatment group. No such correlations were seen in the sham group, and the correlation coefficients between active and sham treatments were significantly different (both p = 0.04).

Since bipolar depression potentially has a different aetiology from unipolar depression, we conducted auxiliary analyses to examine whether including patients with bipolar depression affected the results. When excluding bipolar patients, the correlation between changes in GABA levels in the dACC and changes in MADRS-S scores remained significant (r = 0.64, p = 0.02) in the active group. The correlation analysis of baseline BP_ND_ in the nucleus accumbens and changes in MADRS-S scores without the bipolar patients resulted in a slightly lower correlation coefficient (r = 0.57) and was just above the significance level (p = 0.051). The results in the sham group remained unchanged.

The changes in MADRS-S scores from pre- to post-assessment were relatively low on average (mean: −4.3 [SD: 8.3]). When examining individual items in the MADRS-S, greater reductions were observed in items 1, 2, 3, and 5 compared to the remaining items. Post hoc analyses of these items revealed significant correlations between changes in GABA levels and changes in items 2 (inner tension) and 3 (sleep) in the active group (ρ = 0.62, p = 0.02 and ρ = 0.65, p = 0.01). Changes in GABA levels were also moderately correlated with changes in item 1 (depressed mood) and item 5 (concentration) within the active group, although these correlations were not statistically significant (ρ = 0.48, p = 0.07 and ρ = 0.50, p = 0.06).

The mean creatine SNR was 214 [SD: 36], and the FWHM was 6.5 Hz [SD: 1.0]), both of which were quite acceptable [49]. Spectra from the active and sham treatment did not differ in the SNR or the FWHM for creatine (pre-treatment acquisition: t = 1.24, p = 0.22 and t = −1.41, p = 0.17; post-treatment acquisition: t = −0.01, p = 0.99 and t = −0.69, p = 0.50). The mean relative residuals were 19.5 (SD: 7.7) for the OFF spectra and 7.9 (SD: 3.1) for the DIFF spectra.

## Discussion

In this first-ever study, combining MRS and [^11^C]flumazenil PET to examine the effects of iTBS treatment for depression, we found a correlation between symptom improvement and a reduction in GABA levels in the dACC after active treatment. We also found that lower baseline GABA_A_-receptor availability in the nucleus accumbens was related to symptom improvement following iTBS, although this result fell just short of significance after the exclusion of patients with bipolar depression. We did not find any change in mean GABA levels, glutamate levels, or GABA_A_-receptor availability after iTBS treatment.

### GABA levels

Our findings support the hypothesis that GABA plays a role in depressive episodes and the antidepressant effects of iTBS. The observation that symptom improvement was associated with reduced GABA levels in the dACC contrasts with previous studies that utilised spectral-editing MRS for GABA detection. However, the regions examined in these studies have been the left dlPFC [13, 16, 17] and a more anterior part of the mPFC [14, 15]. These studies have yielded inconsistent results, with some reporting an increase in mean GABA levels after rTMS [13–15], while others found no significant changes compared to baseline [16, 17]. In addition, some of these studies suggest that increased GABA levels are linked to treatment response following rTMS [13, 14], while others report no such correlation [15–17]. The relationship between baseline GABA levels and symptom improvement has also been examined in two separate studies without yielding positive findings [16, 17]. The same lack of association was seen in the current study.

Most earlier studies have been open-label, with all patients receiving active rTMS [13, 14, 16, 17]. Moreover, these studies differ from the current study in their use of a 10 Hz treatment protocol over the left dlPFC. Additionally, one study switched to bilateral treatment after 15 sessions for non-responders by introducing a sequential 1 Hz treatment to the right dlPFC [13]. One small recent study had a randomised, double-blind, sham-controlled design and used an iTBS treatment protocol over the left dlPFC [15].

Besides the variation in stimulation parameters, stimulation intensity is another aspect that could explain the differences in results between studies. The target stimulation intensity has been set at 120% in all previously mentioned GABA MRS studies; however, the actual mean stimulation intensity has varied (86.7% [14], 107.1% [13], and 119.6% [16]. Two additional studies did not report their mean stimulation intensity [15, 17]. In contrast, our study aimed for a target treatment intensity of 90% rMT, and the treatment course was prolonged by one day if at least 50% of the trains did not reach the target intensity on a given treatment day.

Overall, the differences in treatment, target locations, and voxel placements compared to previous studies hamper the ability to compare their results with those of the present study. Further, earlier studies have employed daily sessions over 4–6 weeks, which also contrasts with the present study that utilised a prolonged, accelerated protocol of iTBS. It is plausible that changes in GABA levels may vary across different brain regions, depending on the treatment target and the protocol used.

The post hoc analyses may indicate that symptoms of anxiety and sleep issues are more closely linked to GABA levels than other depressive symptoms. Sleep problems may stem from anxiety due to difficulties in relaxing, potentially making the items related and having a common pathophysiology. However, these are exploratory analyses subject to multiple correlations and should be interpreted with considerable caution. Depressed mood and difficulties with concentration also exhibited a moderate correlation to changes in GABA levels, albeit not statistically significant. Given that these four symptoms together represent core characteristics of depression, the presence of a subset of symptoms more closely associated with GABA may seem less probable.

### Glutamate levels

We found no difference in glutamate levels after treatment with iTBS. This is consistent with several earlier studies [14, 17, 24], although there are also findings of increased glutamate levels following rTMS [16]. Notably, these studies targeted the left dlPFC with rTMS, and most have examined the neurotransmitter levels in the same dlPFC region [16, 17] or a voxel within the mPFC [14]. However, one study included a dACC voxel comparable to ours [24]. Even though this study indicated that improvements in depressive symptoms could be correlated to changes in glutamate in the dlPFC, there were no changes in glutamate levels in the dACC [24]. Similarly, symptom improvement was associated with a reduction of dlPFC Glx in another study, but we could not find any correlation between the improvement of depressive symptoms and glutamate changes in the dACC [17].

We also found no association between baseline glutamate levels in the dACC and the antidepressant effect following rTMS, which aligns with earlier findings in the dACC [24]. However, correlations between lower baseline glutamate/Glx levels and reduced depressive symptoms have previously been observed in the right dACC and the left dlPFC after rTMS [24, 50]. In contrast, one study reported that higher baseline glutamate levels in the left dlPFC correlated with improved depressive symptoms following rTMS [17].

Taken together, comparisons between studies that analyse glutamate/Glx levels after rTMS are hampered by the same problems with different study designs seen with GABA.

### What does MRS measure?

MRS can measure changes in metabolite concentrations at a millimolar level but cannot provide estimates of changes in synaptic activity, which occur at a micromolar level. Both glutamate and GABA exist in metabolic and neurotransmitter pools within their respective neurons. These pools are in continuous exchange, and any of the pools can serve as a source for neurotransmission [51]. Levels of GABA do not correlate directly with excitatory activity, and changes in GABA levels primarily reflect fluctuations in the engagement of the GABAergic cell compartment. Specifically, as GABAergic cells become more active, GABA levels rise. However, metabolic GABA is related to the levels of extracellular GABA, which affects local tonic inhibition [52]. Consequently, total GABA levels serve as a better marker of GABAergic tone than a direct measure of inhibitory activity [51].

Considering this, our finding that reduced GABA levels correlate with symptom improvement could indicate that alleviation of depression is related to reduced GABAergic tone. This could be consistent with the increased rMT found in depressed individuals, as rMT is believed to be influenced by tonic inhibition [53, 54]. Furthermore, left hemisphere rMT has been demonstrated to decrease following successful rTMS treatment for depression [55].

### GABA_A_-receptor availability

GABA_A_-receptor availability in the context of rTMS for depression has not been examined earlier; however, there have been a few studies investigating GABA_A_-receptor availability in depression disorders [18–20]. Most of these studies did not find any significant differences in GABA_A_-receptor availability between depressed patients and healthy controls [18, 19]. Notably, a study using [^123^I]iomazenil SPECT demonstrated a widespread increase in cortical GABA_A_-receptor binding in patients with depression after successful electroconvulsive therapy [21]. A corollary suggestion is that rTMS may similarly influence GABA_A_-receptor binding. Although our present study did not show a mean change in GABA_A_-receptor availability, we did find a correlation indicating that lower baseline GABA_A_-receptor availability in the nucleus accumbens predicts improvement in depressive symptoms. It is possible that lower GABA_A_-receptor availability reflects reduced inhibition on efferent projections from the nucleus accumbens, which, in turn, may facilitate modulation by rTMS on the dACC and frontostriatal loops involved in depressive symptomatology.

### Limitations

Some limitations of our study have to be addressed. The sample size may be too small to reliably detect differences in neurotransmitter levels with MRS compared to the sham group. Sample sizes in most previous studies examining GABA and glutamate levels after rTMS have ranged between 20 and 30 participants, which is likely still on the low side and contributes to the inconsistent results in these studies [13–17, 24]. However, a recent sham-controlled study of iTBS over the dlPFC, which only had post-treatment MRS data for six participants in each arm, found a significant increase in GABA compared to the sham-iTBS group [15].

It is possible that the iTBS applied at 90% foot rMT, administered twice daily for 10 consecutive days, was too short and not effective enough to reach the full potential of alleviating depressive symptoms. This may have reduced the likelihood of detecting significant changes in neurotransmitter levels and potentially led to underestimation of GABA changes. Previous research has demonstrated that GABA levels are lower in depressed patients compared to healthy individuals [11]. The participants in this study were compared to healthy controls in a previous study, where no difference in GABA levels was found [19]. The absence of reduced baseline GABA in our participants may have hampered the antidepressant effects of iTBS and prevented observable metabolite changes in the present study.

Utilising the self-rated version of MADRS may have diminished the reliability and sensitivity of symptom changes and impacted the strength of the correlations examined.

Although pharmacotherapy remained unchanged during the study, it may still have influenced metabolite levels. However, this potential effect is mitigated by the randomisation into sham and active treatment arms. Substance use disorder was an exclusion criterion, but other comorbid diagnoses were accepted. Comorbidities and including bipolar depression, may have increased the heterogeneity of the sample. Nonetheless, the impact of this on metabolite levels should be reduced by randomisation to treatment arms.

We derived glutamate from the MEGA-PRESS OFF spectra, which do not utilise standardised MRS parameters for glutamate estimations. Nevertheless, the OFF spectra still seem to provide a reliable estimate of glutamate [38].

## Conclusion

In this study, we observed that when iTBS improved depressive symptoms, it was accompanied by a decrease in GABA levels in the dACC, indicative of reduced GABAergic tone in this brain region. This finding contrasts with most earlier studies, which may be attributed to the varying rTMS protocols employed. Furthermore, the findings indicate that low baseline GABA_A_-receptor availability in the nucleus accumbens can be related to symptom improvement. This may partly explain the individual variation in symptom improvement and highlights a potential predictive role for frontostriatal GABA_A_-receptor availability in depression treatment with dmPFC iTBS.

## Supplementary information

Table MRSinMRS

## Data Availability

Due to ethical and legal constraints, data cannot be shared. For further questions, please reach out to the corresponding author.
