# Supplementary material for: Alterations in gamma-aminobutyric acid and glutamate neurotransmission linked to intermittent theta-burst stimulation in depression: a sham-controlled study
Source: Transl Psychiatry. 2025 Apr 8;15:133. doi: 10.1038/s41398-025-03371-x (PMC11978943; doi:10.1038/s41398-025-03371-x)
Supplement: Supplementary file 1 — Table MRSinMRS [file 41398_2025_3371_MOESM1_ESM.docx]

**Supplementary material 1**. MRSinMRS checklist. (Lin et al. Minimum Reporting Standards for in vivo Magnetic Resonance Spectroscopy (MRSinMRS): Experts' consensus recommendations. NMR in Biomedicine. 2021;e4484).

| 1. Hardware |  |
| --- | --- |
| a. Field strength [T] | 3 T |
| b. Manufacturer | Philips |
| c. Model (software version if available) | Achieva dStream |
| d. RF coils: nuclei (transmit/ receive), number of channels, type, body part | 32 channel ^1^H head coil |
| e. Additional hardware |  |
| 2. Acquisition |  |
| a. Pulse sequence | MEGA-PRESS |
| b. Volume of Interest (VOI) locations | Dorsal anterior cingulate cortex |
| c. Nominal VOI size [cm^3^, mm^3^] | 4 x 4 x 2 cm^3^ |
| d. Repetition Time (TR), Echo Time (TE) [ms, s] | TR 2000 ms, TE 68 ms |
| e. Total number of Excitations or acquisitions per spectrum | 320 total averages with 160 averages per sub spectrum, collected in 40 groups with each group containing one unsuppressed water line followed by four pairs of water suppressed ON-OFF spectra. |
| f. Additional sequence parameters | Spectral width: 2000 Hz  Number of spectral points: 1024 points  14 ms long GABA-editing RF pulses at 1.9 (ON) and 7.5 (OFF) ppm. Bandwidth of editing pulses 150 Hz. |
| g. Water Suppression Method | Philips “excitation” method. |
| h. Shimming Method, reference peak, and thresholds for “acceptance of shim” chosen | Philips PB (pencil beam) first-order shimming. The unsuppressed water signal was used as the internal concentration reference. Shimming was accepted for water line FWHM < 20 Hz. |
| i. Triggering or motion correction method  (respiratory, peripheral, cardiac triggering, incl. device used and delays) | N/A |
| 3. Data analysis methods and outputs |  |
| a. Analysis software | Osprey 2.5.0 – develop Oct 2023 |
| b. Processing steps deviating from quoted reference or product | Probabilistic spectral registration |
| c. Output measure | Institutional units |
| d. Quantification references and assumptions, fitting model assumptions | Default basis set  Osprey separate fitting for DIFF and OFF spectra.  Baseline knot spacing 0.55 ppm |
| 4. Data Quality |  |
| 1. Reported variables   SNR: max Cr/noise SD  FWHM: the average of the FWHM of the data and the FWHM of a lorentzian fit calculated from the OFF subspectrum | SNR (Cr): 214 +- 36  FWHM (Cr): 6.5 +- 1.0 Hz |
| b. Data exclusion criteria | *None* |
| c. Quality measures of postprocessing model fitting  Mean Relative Amplitude Residual (Residual/Noise) | OFF: 19.5 +- 7.7  DIFF: 7.9+- 3.1 |
| d. Sample Spectrum |  |
